# Living on the edge - circadian habitat usage in pre-weaning European hares (*Lepus europaeus*) in an intensively used agricultural area

**DOI:** 10.1371/journal.pone.0222205

**Published:** 2019-09-09

**Authors:** Ulrich Voigt, Ursula Siebert

**Affiliations:** Institute for Terrestrial and Aquatic Wildlife Research, University of Veterinary Medicine Hannover Foundation, Hannover, Germany; Texas State University, UNITED STATES

## Abstract

Over the last decades, the European hare (*Lepus europaeus*) has become the subject of many interdisciplinary studies due to the sharp Europe-wide population decline. In European hares, the first stage of life until weaning and the subsequent dispersal have been sparsely studied, in particular, habitat selection, movements and survival rate, as juveniles´ precocial lifestyle is dominated by concealment, motionlessness and inconspicuousness. In this study, free-living juvenile European hares (leverets) were detected systematically by thermography (n = 394), radio-tagged or marked (n = 122) from birth until the fifth week of life to research their habitat usage and pre-dispersal movements. The day-resting places and night locations, as well as the distance moved by leverets with aging, were evaluated by generalized linear mixed effect models. In addition, the habitat preference was assessed by a conservative use-availability analysis. Up to the fifth week of life, 30.5% of all leverets used cultivated areas in the daytime. In contrast, the remaining 69.4% animals inhabitated linear or small planar structures in the daytime, with the edges of field tracks, hedges and some ruderal structures clearly being preferred. At nighttime, 93% of all juveniles, which occupied linear structures in the daytime, used the adjoining fields up to 20 m away from the next linear structure. Nocturnal distances of more than 60 m to the next edge rarely occurred before the end of the pre-weaning phase. The time of day and age have a significant influence on the distance moved by juvenile hares. With increasing age, leverets moved less during the day and roamed further at night. The results are largely consistent with the behavioral patterns found in the few previous studies on pre-weaning European hares and show the importance of hiding places for leverets in early life stages. This study should contribute to a better understanding of behavior in juvenile life-history stages of European hares that may help to identify vulnerable phases in their lifecycle. In addition, the findings can refine existing population models and improve conservation efforts.

## Introduction

According to numerous studies over the last decades, the European hare (*L*. *europaeus*) has become an endangered species in many central European countries [1–4]. In connection with the fact that this threat is apparent and the hare is known as an important game species throughout its historical range, conflict arises between protection and sustainable use. The primary cause of long-term decline in hare populations throughout Europe is widely assumed to be the intensification of agriculture [5], particularly by farmland management practices [6]. Consequently, this persistent development results in the loss of crop and landscape diversity [7, 8], and ultimately in reduced habitat quality [9]. In addition, other factors such as precipitation, low environmental temperatures or predation play a lesser role and can be considered as secondary factors [3, 10].

Over the past decades, many European studies have been performed on adult and subadult European hares as well as related species. The findings significantly improved the understanding of the ecological relationships of this species in today's cultural landscape, e.g., habitat associations [8, 11], reproductive biology [12, 13], energetics and nutrition [9, 14], activity patterns [15, 16], agricultural practices [17, 18], survival, predation and hunting [19–23], and, among others, also diseases [24, 25]. However, comparatively little is known about wild juveniles during the period of life from birth up to the stages of weaning and dispersal in evermore drastically changing agricultural landscapes.

In Central Europe, reproduction of *L europaeus* usually lasts from January to October [1]. The females give birth three to four times a year, with the litter size varying between one and four depending on the season [23]. The average size of yearly born juveniles is estimated to be between nine and 11 per female [23, 26].

In contrast to many other lagomorphs [27], the genus Lepus invests as a precocial species in a rapid growth of the juveniles to escape the risk of predation, which is considered to be one of the most important mortality factors in the early stages of life [22, 23, 28]. The prerequisites for a fast juvenile development are high-quality food resources [10] for the mothers, which passes the energy on to the leverets via milk, and various anti-predation strategies among the juveniles and the females. The latter includes morphological traits and especially behavioral mechanisms, such as the entire nursing behavior, which is characterized by a minimum of contact time between the female and litter without any additional parental care, as well as the independent search for safe resting places until the next suckling act [29].

It has been shown that leverets, i.e., juvenile hares from birth until weaning, become active shortly after sunset, leaving the day-resting place and gathering with their littermates to be nursed by the female about two hours after sunset, with little variation in timing [30, 31]. After an average three-minute suckling [32], this temporary community breaks up. At the latest, the leverets return to their hiding place for the remainder of the night and stay without parental care until the following nursing act. This procedure is repeated until weaning in the fifth week of life, with the exact position of the nursing place rarely shifting. This separated lifestyle has been the focus of only a few studies on the European hare to date. However, it has been described more often for the related species *Lepus americanus* [33, 34] and *Lepus timidus* [35].

There is a close relation between the place of birth and the suckling place [36] as well as the consecutive day-hiding places of the leverets during the suckling period [30]. There is some preliminary evidence of dispersal after weaning [36, 37], but the questions concerning daily habitat use, survival and causes of death in the first weeks of life remain largely unanswered.

The reasons for this are likely to be found in the challenges involved in studying an animal in the wild whose lifestyle is shaped by inconspicuousness, motionlessness and use of shelter, reducing the detection probability and ultimately decreasing the risk of predation in the first weeks of life. The availability of thermal imaging technology over the past 15 years has resulted in new opportunities in wildlife research, particularly in detecting of concealed-living animals. Thus, this technique is used to detect leverets systematically as soon as the days´ resting place was left for a short time to be nursed by the female.

The present study aimed to fill gaps in our current knowledge by characterizing the habitat preferences of leverets in a circadian context and investigating the early movements within the first weeks of life. Based on previous research, this study hypothesized that leverets select rich habitat structures for any kind of coverage. Furthermore, it was expected that the distance moved by leverets did not increase with aging up until weaning in the daytime and vice versa at nighttime. This may contribute to a better understanding of hares´ early ecology and population dynamics, and it could lead to an improved farming and landscape management.

## Materials and methods

### Study area

The study was performed from 2004 to 2010 in the Hildesheimer Boerde landscape, which belongs to the Central European loess zone, a part of the North German Plains (Study area: approx. N52.244118°/E10.116405°, S1 Fig). Hildesheimer Boerde is a slightly undulating, little structured, large-scale agricultural landscape and is mainly characterized by cultivation of winter cereals (44%), sugar beet (25%), maize (16%) and potatoes (up to 5%). On a seven-step scale, soil fertility and yield capacity achieve the "extremely high" category, the best value [38]. Due to this intensively managed agriculture, forests are completely absent and grassland accounts for 5.7% (105 ha) of the total study area (1842 ha). Other wooded landscape elements, such as hedges, copses and lines of trees, are slightly more frequent but limited to road sides and field paths and account for 0.72% (13.5 ha) of the investigated area. An Atlantic climate prevails, with an average annual precipitation of 646 mm and temperature of 10.4 °C. The overall size of the study area was approximately 1,800 hectares. The population density estimated by spotlight counting [39] averaged from 20 to 52 per km^2^ in spring and 30 to 68 hares per km^2^ in the fall. Nationwide, the study area featured a medium to high density of hares [40]. Hares are hunted once a year, mostly in the first two weeks of December.

### Detection of leverets

In the lapse of time between leaving the day's hideout and returning to it, young leverets can be detected by an observer. A thermography imager camera (Palm IR 250-D; Raytheon Company, US) equipped with a 100 mm telephoto lens was used to detect leverets. The 320x240 pixel microbolometer detector array was able to differentiate thermal radiation between objects and their direct surroundings of 0.2 °C, so warm objects like mammals could be detected easily. Although the thermal imaging camera ensures reliable detection of small and distant warm objects, an instant identification of different species, e.g., leverets, hedgehogs or rats, was difficult due to the low resolution. Behavior, size and shape of the detected object, as well as the experience of the observer determined a worthwhile approach in most cases. Once a warm target had been located, the animal was approached on foot until identification by flashlight was possible.

Two search techniques were performed depending on the field size, the height of vegetation and the associated reduced detectability of warm objects at ground level at a greater distance. Usually, the landscape was scanned from the back of a pick-up truck driven along field tracks and across untilled fields, e.g., bare soil, stubble fields or collapsed cover crops. Searching from both sides of the truck and using the elevated position of the camera at about 3.5 m above the ground resulted in a more favorable detection performance and probability, as the latter strongly depends on the elevation angle for distant objects. Alternatively, fields were crossed on foot and searched using a GPS-guided meander-like pattern at a maximum distance to each transect part of 200 m. The combination of the two variants resulted in an almost complete scan of the fields.

The investigation was performed in two seasons per year because detection of warm objects in dense crops and other vegetation is virtually impossible. Season I incorporated data from early February to late May, season II incorporated data collected after cereal harvest, from mid-July to the end of September, which coincided with the end of the hares´ main reproductive phase. The searches were conducted exclusively at night, beginning 60 minutes after sunset at the earliest, and lasting between four and seven hours per night depending on the season.

### Capturing, handling, tagging and age determination

Capturing, handling, tagging and radio-tracking of juvenile hares were conducted under a permit held by the Lower Saxony Institute for Consumer Protection and Food Safety (LAVES, Dept. 3 Animal Health, permit number: 33.12-42502-04-16/2083). Leverets were caught directly by hand since they showed no defensive behavior nor escape response, and larger juveniles were captured with a landing net (1 m in diameter) fitted with a 5m handle. To avoid further disturbance in the vicinity of the capture site, the subsequent procedure was carried out at a distance from that site by transporting the leveret in a small box. The capture site was digitally saved in a handheld GPS by automatically positioning for ten minutes at one second intervals, which lead to a horizontal accuracy of 1.7 m ± 0.3 m. Capturing took place one and a half hours after sunset at the earliest.

During handling, care was taken to provide a quiet environment, and the animal's head was always covered. In addition to the body weight, further biometric values such as ear length, hind foot length, skull width and length were recorded.

Depending on the body weight, juveniles were fitted with 1.4 g (Model PIP, 150 MHz, 12 cm antenna; Biotrack Ltd., UK) and 4.0 g (433 MHz; AWEK fl-electronic GmbH, Germany) adhesive VHF-transmitters, which weighed less than 2% of the body mass. The glued transmitter became detached after a few days or weeks from the animals’ fur. For subsequent recognition after the transmitter had dropped off, each captured animal was provided with a marker in the left ear (Model 1005–3; National Band Tags & Co., US). Radio-tagging took place between 2007 and 2010, and only one animal per litter was tagged.

A surgical adhesive (EPIGLU®, Meyer-Haake GmbH Medical Innovations, Germany) was used for attaching the transmitters. The hair along the shoulder blades and neck of a juvenile was parted before applying some glue to the underside of a transmitter. The transmitter was then carefully glued onto the undercoat but not to the skin. Afterwards, the juvenile’s hair was folded over each side and over the top of the transmitter and was glued once more to these sides while the antenna rested along the length of the animal’s back and was directed slightly upwards. After a recovery time of approximately 50 seconds, the leveret was again placed in the capture box and immediately released at the initial capture site. To reduce the effect of repeated disturbance, tags were never re-glued to the same animal. Juveniles were held captive for a maximum of nine minutes.

The age at first capture was estimated from the nearly linear allometric relationship between skull length and weight [41]. To maintain accuracy of this calculation, some animals were captured and measured two to three times. Otherwise, the age of relocated animals was determined based on the time lapse since the initial detection.

### Monitoring leverets

Since juveniles remain at their habitat and show no pronounced dispersal behavior in the first weeks of life, the telemetry technique ‘homing’ [42, 43] was applied as the ultimate method for tracking leverets. Telemetry was performed by a 3-element yagi antenna and a receiver suitable for the used frequency type (150 MHz, SIKA, Biotrack Ltd., UK; 433 MHz, FME 434, AWEK fl-electronic GmbH, Germany). Each juvenile was located up to five times a week at different times during the day and night up until its death, the falling off of a transmitter or transmitter failure was confirmed. The signal was always localized to a half square meter, and the position was stored in a handheld GPS. Visual confirmation was often omitted, especially in dense vegetation, to avoid disturbing the leverets or attracting predators. In such cases, life status was determined retrospectively by the well-documented exact positions, their changes over time or entry of a final stage. In the event of a signal loss, the tags were searched spaciously by a truck-mounted telemetry system at an antenna height of 8 m to increase detection probability. The last location referred to as ‘alive’ was additionally checked by thermography several times at night to reduce the likelihood of a transmitter failure. If neither the transmitter nor the animal was found again, the animal was assumed dead. The same classification applied to the finding of carcasses or tissue parts such as bones, organs, fur with skin remnants or blood. Cases of signal loss and re-sighting the animal alive were noted as technical failure.

### Data processing and analysis

The entire study area was mapped, and each position of a leveret was assigned to one of eleven habitat classes (Table 1), ‘location time’ (night versus day), ‘edge distance’ (the distance to the next edge) and ‘distance moved’ (the distance between consecutive positions of the animals). All spatial calculations were performed using ArcGIS Desktop 10.6.1 (ESRI Inc., US). Waters, buildings and asphalted roads were not included in the analysis because they cannot be used by leverets as day-resting places. A web-based calculator [44] was used to assign the time of the hares’ detection between the beginning of civil twilight in the morning and the end of civil dawn in the evening to day locations and vice versa to night locations.

All data preparation and analyses were modeled in R 3.5.2 [45] by using the following R packages: lme4 for statistical models [46], mumin for calculating pseudo-R-squared values for mixed-effect models [47] and ggplot2 for the graphical output [48]. Model selection was accomplished by AIC comparisons using maximum likelihood estimations.

**Table 1:** Defined habitat classes aggregated from single habitats and separated by edges or areal characteristics.

Geometric habitat properties	habitat class	habitat class abbreviation	description of individual habitats
in edge habitats (non-agricultural)	ditches-grassy	DG	ditches (pipe culverts included) and grass strips (apart from adjacent farm tracks)
	fallow-storage	FS	barns, fallow land, ruderal sites surrounding winddriven power generators or fences or human-related storage yards, silage
	copses-hedges	CH	wooded habitat such as trees and shrubs that stand together in groups and whose geometry is linear (hedges) or planar (copses)
	residential associated	RA	allotments, gardens, areas surrounding buildings, recreational areas (sports, riding etc.)
	road side ditch	RD	strips up to 5 meters wide along each side of the road; area of road asphalt is excluded
	field tracks	TR	asphalted, gravelled or unmade farm tracks with grassy double wayside or with one-sided grassy strips and a ditch on the other side
outside edge habitats (agricultural)	pasture	PA	pastures, meadows
	crops (four buffer classes)	C20 C60 C100 C>100	arable land with crops and non-tilled fields (divided into buffer classes starting from the adjacent edges: 0–20 m (C20), 20–60 m (C60), 60–100 m (C100) and >100 m (C>100))

#### Habitat preferences

The selection of day-resting places and night locations by leverets was evaluated by a use-availability analysis [49] in accordance with Cherry [50]. This method combines the Chi^2^ goodness-of-fit test [51] at a significance level less than 0.001 with Bailey’s simultaneous 95% confidence intervals [52] for the observed usage value for each habitat class. The simultaneous Bailey intervals [52] are based on large-to-moderate sample properties, but are fairly robust and not particularly sensitive to small sample sizes [53]. This method is considered reliable when comparing availability of each habitat type to the observed habitat usage [42]. Additionally, the initial use-availability method [49] did not increase the type 1 error rates, particularly with regard to smaller positive values for each utilization habitat category [54], thus providing confidence in the present analysis.

Some listed single habitats (Table 1) had to be allocated to habitat classes to meet the requirements of cell frequencies close to zero [52]. To this end, a 200 m buffer was placed around the corresponding localizations according to season and time of day. Both the number of localizations and the area percentage of the habitat classes were summarized within the respective buffers. The size of the buffer resulted from the maximum circadian radius of movement, which was averaged among all leverets in the first weeks of life and is supported by the findings of a study on natal dispersal in young hares [36]. Leverets preferred a habitat class if usage and the corresponding confidence limit was higher than in the expected available habitat class percentage. In contrast, a class was avoided if the expected available value exceeded the confidence limit of the one actually used. Accordingly, the term ‘equally use’ applied if the expected value met the confidence limits.

#### Occupancy of linear habitats

To analyze the relationship between the usage of linear or linear-like habitats, hereafter termed ‘edge’, and the diurnal location type and increasing age of leverets, a generalized linear mixed-effect model (GLMM) was performed by binominal distribution.

The response variable was in the edge habitat (1 = in the edge habitat versus 0 = outside the edge habitat; for attribution see Table 1) was fitted with the fixed effects of ‘location time’, estimated leveret´s age and the random effects of individual animals and the corresponding year. A set of models with additional fixed and random effects like gender (male or female) and season (seasons I and II) was generated. However, it turned out that these variables did not improve model fit based on AIC and they were therefore excluded. The full model was used without interaction term. The analysis was performed by including only radio-tagged leverets with an age up until weaning, i.e., younger than 35 days.

#### Distance moved

For contrast, a second model was used to assess the distance moved by juvenile hares as they grew older. To model this relationship, a linear mixed-effect model (LMM) was performed on log-transformed distances between consecutive positions (distance moved) as a response variable. The calculation and classification thereof were based on the distance between day-day fixes (day distance) and between day-night/night-day/night-night fixes (night distance). The distance between consecutive day-night positions and vice versa was very likely to had been arisen at night, since leverets started their activity after dark at the earliest [29]. Therefore, the allocation of the day-night-fixes to the category of 'night points' was justified. To reduce the large scattering caused by a few locations or animals, hares with an age older than 30 days and fewer than three localizations, so-called outliers, were excluded from the analysis. The factor ‘location time’ (day or night) and the variables age and distance moved were entered as fixed effects. The individual animal was integrated as random effect. In a first exploratory step with some sets of variables, the seasons proved not to influence the quality of the model and thus were excluded.

The full model designed with a non-random intercept was compared to the null model by the likelihood ratio test using the one-way Anova test. While no exact equivalent to the R^2^ of linear regression exists, a pseudo R-squared for generalized mixed-effect models was used to assess the model fit.

## Results

### General results

In total, 394 individual juvenile hares were detected by thermography during the study period, of which 70% were found in season I and 30% in season II. Of these, 122 animals were equipped with radio-tags between 2007 and 2010 (Table 2).

**Table 2:** Number of detected and radio-tracked animals.

	2004	2005	2006	2007	2008	2009	2010	Total
Season I	34 (-)	32 (-)	57 (-)	37 (6)	50 (32)	36 (22)	30 (21)	276 (81)
Season II	10 (-)	13 (-)	17 (-)	24 (5)	26 (13)	13 (12)	15 (11)	118 (41)
Total	44 (-)	45 (-)	74 (-)	61 (11)	76 (45)	49 (34)	45 (32)	394 (122)

The number of juveniles detected by thermography is given for each year and season; the number of radio-tracked animals is given in brackets.

Depending on the weather conditions, size of the leverets and the surface structure of the field, juveniles were detected by thermography between 4 and 247 m (an average of 126 m, SD = 50.8) regardless of the season. The estimated age at first detection for all animals ranged from birth to approximately 100 days with a median of six days (SE = 0.506, SD = 9.05), but there was a difference between season I and season II of five versus 11 days, respectively (Mann-Whitney U = 5816.0, n_1_ = 226, n_2_ = 95, p < 0.000 two-tailed; see also S2 Fig). There was no difference in age between those animals without radio tags and radio-tagged animals at the first capture. The retention time, which is defined as the interval from tagging to dropping out or loss of the vhf signal, had a mean of 11 days (SD = 7.7, 95th percentile = 24, a maximum of 50). In conjunction with the capture of older juveniles, this resulted in a period monitored by telemetry until the beginning of the fifth week of life and covered almost the entire period up until weaning. The gender ratio of all captures was balanced (48.3% males with n = 146 and 51.7% females with n = 156).

The birthplaces of juveniles could not be recorded with the exception of two direct observations, one during the day and one at dusk. Of all first detections, 38.0% (n = 122) were registered up to an age of four days, and an additional 15.9% (n = 51) were registered by the end of the first week of life. Given these ages, one can assume that the capture place of the former group was the approximate place of birth. This assumption is supported by the observation that these few-day-old animals did not move away from their place of first detection in the following days.

### Habitat usage

The habitats occupied by leverets were closely related to the time of day. In total, 69.4% (n = 82) of all animals used the in edge habitats and 30.5% (n = 36) the outside edge habitats during the day, whereas at night there were 6.5% edge users and 93.5% non-edge users. However, the following results of habitat usage (S1 and S2 Tables) refer to the number of locations rather than animals recorded up to the fifth week of life.

### Day locations

In general, radio-tagged leverets (radio locations n = 465) were relocated well-concealed at their day-resting sites and usually alone after they had left their place of birth.

**Fig 1:**
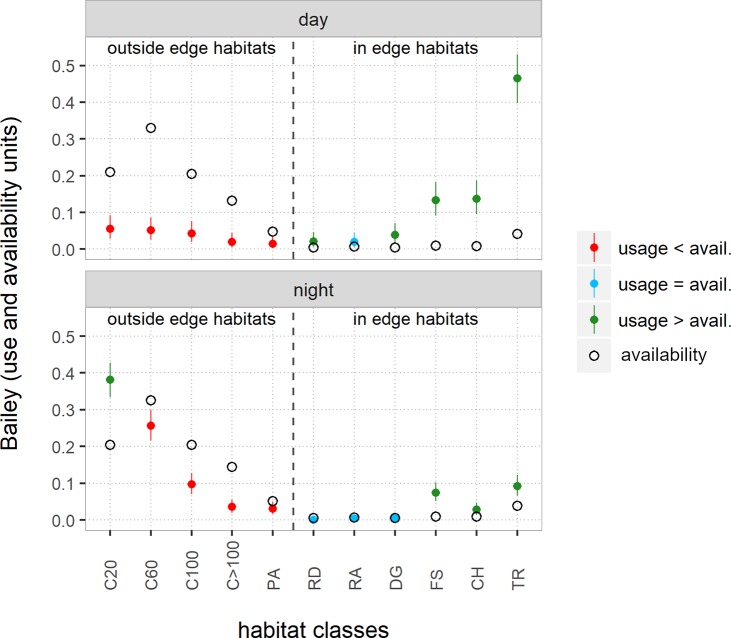
Habitat use-availability analysis [50, 52]. Availability proportions of habitat classes (open circles) and percentage of use by leverets in the daytime (day) and at nighttime (night), each combined for the two seasons (solid circle with whiskers for the Bailey’s 95% confidence limits: red = ‘use < availability’, blue = ‘use = availability’, green = ‘use > availability’). Linear habitat classes (edge) are separated from areal habitats (non-edge, i.e., crop fields and pastures).

Juveniles occupied less agricultural areas than availability of these habitats (Fig 1, day). Equal usage was observed for the habitats residential, road-side ditch and ditches with grass strips (RA, RD and DG). In contrast, small-scale covered structures such as fallows (FS) and linear habitats like hedges, copses and field tracks (CH, TR) were occupied to a greater extent although they were not as prevalent. The latter habitat was by far the most frequently used habitat, according to the Bailey units. The class of ditches with grass strips seemed to be used the least because these were solitary structures far away from field tracks. Therefore, the sum of these habitat areas was comparatively small. In the study area, however, more than 80% of all field tracks included a ditch and always a lateral grass strip, which were therefore assigned to the field tracks class. Leverets used individual shelters repeatedly in the daytime.

### Night locations

In comparison to the daytime, a very contrasting scene emerges (radio locations n = 882, Fig 1, night). The habitat class C20, which represented the closest range around an edge habitat, was used more than its availability. The same usage pattern emerged for all other agricultural areas (C60, C100, C>100) and pasture (PA) but this was less pronounced compared to the daytime pattern. The habitats fallow-storage, copses-hedges and field tracks (FS, CH and TR) showed only slight occupancy, since the leverets stayed in these structures during the day, mainly changes to habitat class C20, but also C60 and C100 at night. The remaining number of juveniles in the classes fallow-storage, copses-hedges and field tracks was related to the control time late at night. These animals had already been suckled and had returned to their day-resting places.

There was no difference in the occupancy pattern for the habitat classes regardless of the analysis was calculated either with the number of individuals instead of the number of radio localizations or separated into seasons.

### Occupancy of linear habitats

**Fig 2:**
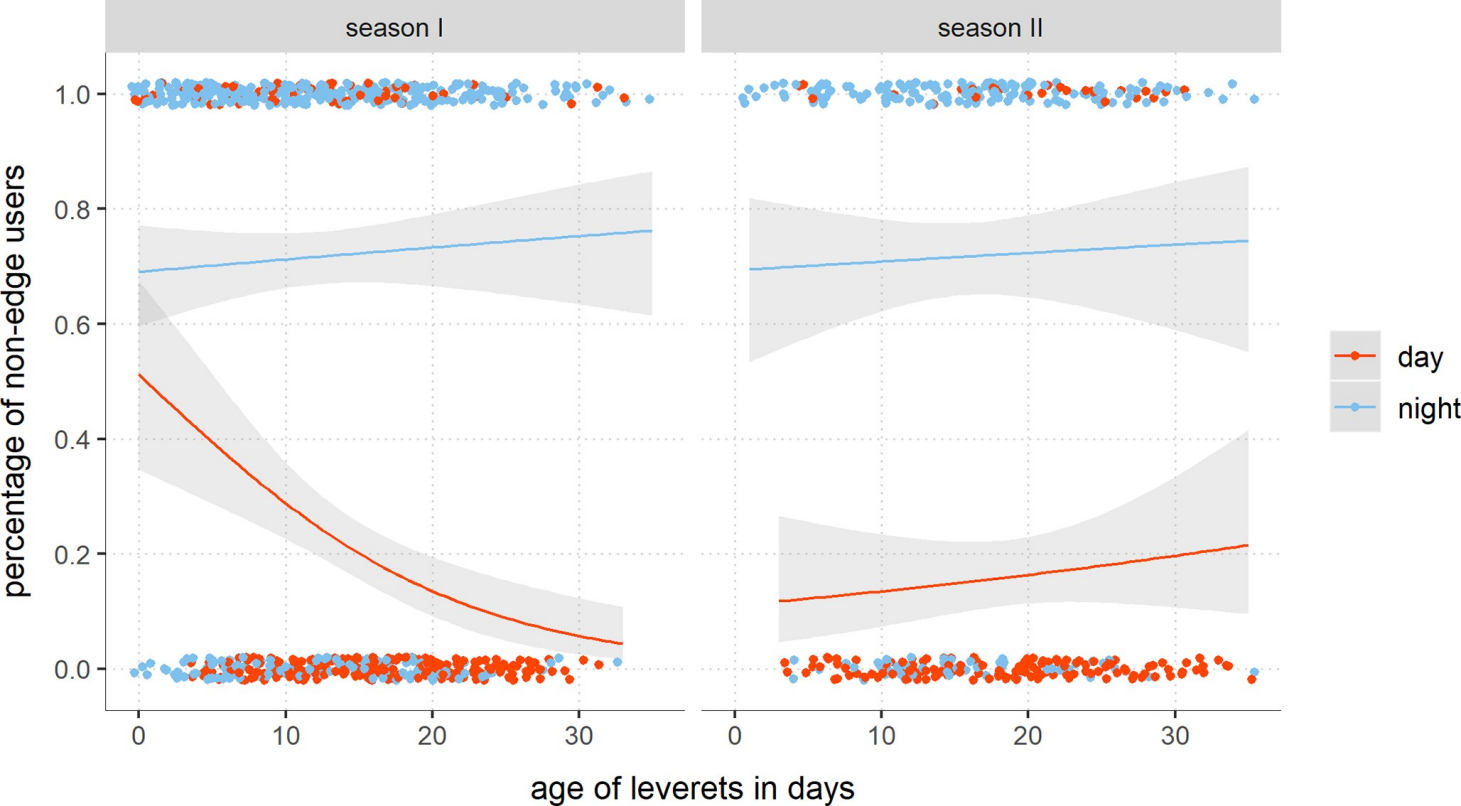
Diurnal and seasonal percentage of leverets using edges. The binomial generalized model shows the functional relationship between increasing age of juvenile hares and the usage of non-linear structures in the daytime (red dots and lines) and nighttime (blue dots and lines). The 95% confidence interval was shaded for day- and nighttimes.

The following results confirmed the aforementioned habitat usage. In contrast, this analysis dealt not with the habitat class, but also with the general influence of the edge on the leverets’ choice of location. For both seasons, it clearly showed that, firstly, juveniles occupied the edges during the day and, secondly, leverets were significantly less frequently at night at these habitats (n_animals_ = 216, n_obs_ = 1032, df.resid = 1027, p < 2e-16, R^2^ full model = 0.801). In season I, 43.6% of all leverets aged up to one week old were found in non-edge related habitats like fields and pastures (Fig 2).

In most cases, these were untilled fields with winter furrows or slightly cultivated ones. In some cereal fields, the seed drill had malfunctioned and as a result had produced double-density rows of seeds in which the juveniles could hide well during the day. About 76% (n = 13) of these hares in open, non-covered habitats were predated within a few days, whereas the surviving leverets actively moved to the neighboring field edges within the first ten days of life. Starting from the second week of life, the proportion of non-edge users was reduced by more than half. Consequently, 80% (n = 18) of all hares used edge habitats during the day up to an age of two weeks. From the third week of life, almost all animals were located at the edges in the daytime, where much coverage was available. This situation changed again between the fourth and fifth weeks of life, when weaning started. In season II, only a few non-edge users were found in the daytime during all weeks of life. In comparison with season I and the first ten days of life, this could be explained by the fact that this age group was comprised of fewer animals. There was no difference in the percentage of outside edges users between the seasons.

### Distance moved

**Fig 3:**
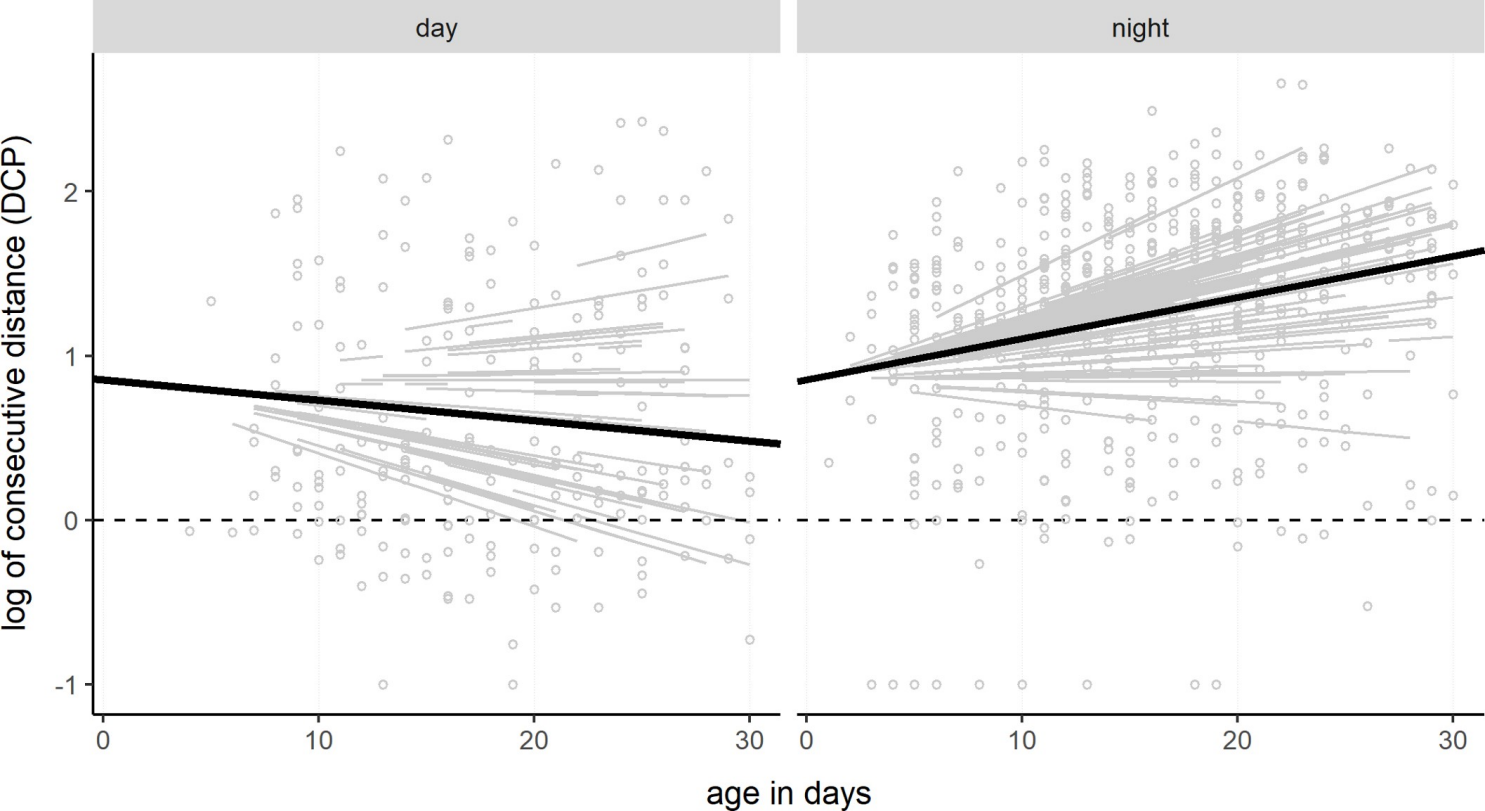
Distance moved by pre-weaning leverets. The log-transformed distances between consecutive day-day and day-night locations are shown depending on the leverets’ age. With increasing age, leverets moved less in the daytime and more at nighttime.

The average distance moved amounts to 19.02 m (n = 211, SD = 42.37, SE = 2.92) for the day-day locations and 38.87 m (n = 542, SD = 48.02, SE = 2.06) for the day-night and night-night locations. The distance between two consecutive localizations during the daytime decreased by 1.03 m per day (log10 = 0.01234, SE = 0.00514) and increased by 1.06 m per day (log10 = 0.02510, SE = 0.00459) at nighttime. Fig 3 illustrates that trend well, although the great variability between the individual animals can be clearly seen. After the litter broke up, radio-tagged leverets were found in 76% of all observations at the same daytime resting place on consecutive days. This is also confirmed by the comparison of the models by Anova, which reveals that the age of leverets and ‘location time’ significantly affected the distance moved significantly (Chi^2^ = 60.111, df = 2, p = 8.851e-14).

In short, young hares moved less during the day with increasing age and roamed further at night. The R^2^ for only fixed effects could be improved from 0.174 to 0.415 by using the full model.

## Discussion

The results are largely consistent with the behavioral patterns found in the few previous studies on pre-weaning European hares [30, 36] and show that leverets select rich habitat structures for any kind of coverage in the daytime.

This indicates the importance of hiding places for leverets in early life stages. In addition, the assumption could be confirmed that the distance moved by leverets did not increase with aging up until weaning in the daytime and vice versa at nighttime.

### Habitat usage, occupancy and moved distance

In the wild, systematic birth observations of hares are very difficult to perform. Births are as little investigated as are the criteria that lead to the selection of a birthplace by female animals [30]. From a biological point of view, and assuming anti-predation strategies in hares, it would be counterproductive if the females gave birth in the open without any shelter. Juveniles would have to move over longer distances to the next hiding place and would thus be more conspicuous. Thus, proximity to edges or structurally rich areas would be preferred as birthplaces. This assumption is supported firstly by the fact that most of the few-day-old hares found in this study were located close to edges. Otherwise, more animals within this age group would have been detected at greater distances to edges. Secondly, most of the night detections were close to edges as well. Thirdly, the nursing site and place of birth are supposed to be close to each other [36]. In contrast, leverets can reportedly move up to 100 m from their daily hiding place to the nursing site within the first ten days of life (*L*. *timidus* [35] and *L*. *europaeus* [36]), which can be extended to a maximum of 250 m by the third week of life [36]. Covering a greater distance takes more time and probably attracts more attention in the environment. This exposure could in turn lead to an increased risk of predation.

Juveniles of the European hare generally leave their location at birth up to an age of three days to find their own daily hiding place at a certain distance from their birthplace and separated from the littermates [29]. An equivalent behavior and an age of one to four days or an average of 2.7 days is given for the snowshoe hare [33, 55]. The leverets gather after sunset once a day for the first four weeks of their lives to be fed by their mothers, but otherwise they receive no parental care.

In addition to the results of the present study, the few existing studies on habitat selection in young animals of different hare species [29, 35, 55] have established that juveniles always actively seek good cover opportunities, e.g., 92% of all snowshoe hares were found under shrubs, clumps of grass, logs or in deadfall during the day [34]. The comparability of those studies with the present study is certainly limited because actual habitat usage depends on the availability or features of habitats in the study area. Overall, the specific habitat type does not seem relevant but rather the existence of well-concealed shelters, which improve survival of the four-week suckling period in which young hares are confined to their habitat.

In contrast to heterogeneous habitats, such as forest borders or unspoilt areas, spaces with cover opportunities are inevitably limited to the edges of paths, ditches or other linear habitats in intensively farmed landscapes featuring large fields, which is also reflected in the results of this study. Nevertheless, leverets could also be found far from the edges in the fields in the daytime, which are poorly provided with adequate shelter compared to the edges. Furthermore, because of their visibility, juveniles were often predated at least in the periods of low to moderate vegetation cover.

The reason for the leverets’ sedentariness in the open habitats during the day may be that they were too young to look for protection in the edges on their own, that suckling did not take place due to some massive disturbances or that, due to suboptimal sowing, there were denser rows of seeds in some cereal fields, which are specifically frequented by juveniles. Thus, the preference for or occupancy of habitat edges or any kind of cover is likely to be influenced by shelter availability.

If there is only poor shelter available, it is to be assumed that it is still occupied, since there is no other option for the juveniles. However, this won´t mean that the shelter is of high quality.

The results document the risk of being killed by agricultural practices in the daytime in the spring, especially on fields with winter cereal crops, which are usually not subject to soil tillage, is low for hares. However, there is a greater risk of mortality while the seed bed is being prepared for sowing sugar beet, maize and summer cereals, especially when work is extended to or after dusk when leverets are waiting to be nursed outside their day-resting places. This is specifically true for few-day-old or few-week-old hares, which are more likely to cower than escape from an imminent threat.

This study’s observation that leverets move less frequently during the day as they grow older confirms the observations and findings already described as well as the principle of hiding during the day and being visible only briefly at night to avoid predation [29, 30, 55].

In contrast, the distances between night-night and night-day locations (night distance) increase with age according to the ‘distance moved’ mobility GLMM analysis. The place of suckling is determined at the outset by the mothers [29, 33, 35]. When shelter at the places of birth or nursing of juveniles born in the field is not available, the young are forced to overcome the distances to the next cover for each suckling act. These results are supported by individual observations with thermography in the present study and in some previous case studies [30, 33, 56] in which leverets show a certain exploratory behavior before and after suckling. The distances covered are generally short. In the present study, the leverets were found or relocated at different times in relation to the suckling act at night, which may have biased the data. Due to the representative sample size, however, these traits are apparent.

Overall, no large values were achieved during the phase of pre-weaning, although a difference and a tendency can be determined in the case of ‘edge distance’ and ‘distance moved’. This was expected because juveniles must behave inconspicuously until they have survived the suckling period due to their precocial system.

From individual observations in this study it is known that from an age of 30 days, the distances between successive day and night locations grew rapidly with a simultaneous increase in the distance to the places of birth and nursing. This period could indicate the end of weaning, which coincides with observations from other studies [29, 32].

Generally, a critical component of population dynamics in birds and mammals is seen in the survival of juveniles to reproductive age. Variation in survival could have significant effects on population growth and viability [57]. Therefore, the survival strategy of the species in connection with the influencing environmental factors in the respective habitat is of existential importance. Most lagomorphs have evolved survival mechanisms adapted to different environmental landscapes and situations [58], but being on the lower end of the food chain the occupied habitat must at least provide high quality forage and adequate cover. Moreover, the rapid and healthy growth process in juveniles is fundamentally related to quantity and quality of nutrition [9, 41, 59]. In fact, mortality is a critical factor in the population growth of European hares [19] or other leporids [60] and may reach as high as 90% among juveniles per year. The maternal strategy in the precocial European hare is hardly shaped to minimize the risk of predation to the young during the most vulnerable stage of life. This leads to a specialized pre-weaning behavior and some other aspects in early life, for instance the synchronous meeting of juveniles, the highly concentrated milk supply [32] for the short infrequent nursing, the minimized time the litter is at any one spot, the time of suckling as well as the independent and widely dispersed hiding places of juvenile littermates far away from the nursing site. As habitat quality affects individual quality, it is consequently of great importance to identify these links between habitat composition and the resulting effects like usage and survival rates.

### Detection, capture and handling

In adult European hares, changes in spatial distribution and movement rates due to increasing predation risks have been observed [37, 61]. A human-related predation that may affect species behavior and distribution in various ways is described for hunting [62], and one can assume the same for any kind of manipulating animals. Ultimately, this study fails to elucidate the extent to which capturing, tagging and relocation had an influence on the choice of the daytime resting places or the overall moved distance. However, in a study with the mountain hare (L. timidus), the impact of manipulating was not considered significant [35]. With the exception that the nursing place becomes inaccessible, this locality was fixed and not changed by the female [31]. Thus, it has to be assumed that the leverets´ behavioral pattern regarding the search for cover changed only slightly as a result of the study manipulation. In addition, agricultural processing, predation on litter mates or the mere presence of predators may have lead to a change in behavior, potentially resulting in more variance in the data.

Despite the availability of metric values of the exact distance of each location to the next edge are available, the used model on linear habitats should be sufficient in principle because, from a biological point of view, it should only emphasize the use of the edge and not the exact distance.

Although the applied method of thermography is quite time-consuming and an expensive acquisition, it is nevertheless suitable for systematic detection of leverets in wild populations a few days after birth. This was confirmed by the found distribution of the age at first detection in the present study. It is obvious that the denser the vegetation, the fewer animals can be detected. Thus, the usability and significance of this method are limited to periods of low vegetation, and it remains unclear whether the ascertained behavioral pattern changes with the thickening of vegetation and complete covering of the fields, i.e., to what extent the field track edges no longer serve as daytime hiding places. This knowledge gap could only be closed by experiments such as those carried out on pregnant snowshoe hares in temporary pens [33, 34].

## Conclusions

The preference of leverets to hide in cover-rich structures, especially in field track edges, ditches and fallow-like strips, emphasises the importance and quality of these habitats. Due to of the lack of diversity in today's agricultural landscape, which is the main cause for the long-term decline in the European hare’s population, these structures should be implemented more readily. According to the ecological focus areas (EFA) of the Common Agricultural Policy (CAP) or other national agricultural funding schemes, creating and designing new areal or strip-type habitats with flowing transitions between fields and their edges could improve the habitat quality and probably the survival rates of juvenile or adult hares. Even the protection of existing structures like field tracks or ditches, such as avoidance of driving along or mowing during the reproduction phase, could be important elements to support an increasing habitat quality.

Based on these field experiences, future research should combine thermal imaging technology with drone technology to extend the entire coverage period and the search to include denser vegetation. In addition, this would offer the opportunity to verify the study area more randomly, which would ultimately improve the prerequisites for statistical methods. However, legal restrictions as well as incidents involving the public due to drone use could hinder future research.

This study aims to further the understanding behavioral patterns in juvenile life-history stages, which may help to identify vulnerable phases in the lifecycle of European hares for identifying major causes of death, to refine existing population models and to improve conservation efforts.

## Supporting information

S1 Fig Location of the study area. A) Base map with location of the study area (red dot) in the federal state Lower Saxony (grey shading) in Germany. B) The elevation map shows the geographic position of the study area (red dot) in the North German Plains. The study area is situated in the natural region ‘Hildesheimer Börde’ (red hatched area), which is characterized by an intensively used agricultural landscape. For orientation some cities (grey squares) are shown.
(TIF)

S2 Fig Estimated age of leverets at first detection. The violin and box plot prove that thermography is a useful tool for detecting leverets within their first days of life. 75% of all juveniles found were less than ten and 17 days old in season I (red violin = spring) and season II (blue violin = summer), respectively.
(TIFF)

S1 Table Use-availability analyses for aggregated habitat classes and both seasons in the daytime. The type of use is classified as preference/occupancy (+), equal use/no selection (=) or avoidance/non-occupancy (-).
(DOCX)

S2 Table Use-availability analyses for aggregated habitat classes and both seasons at nighttime. The type of use is classified as preference/occupancy (+), equal use/no selection (**=**) or avoidance/non-occupancy (-).
(DOCX)
