# Supplementary figures and images for: Living on the edge - circadian habitat usage in pre-weaning European hares (Lepus europaeus) in an intensively used agricultural area
Source: PLoS One. 2019 Sep 9;14(9):e0222205. doi: 10.1371/journal.pone.0222205 (PMC6733508; doi:10.1371/journal.pone.0222205)

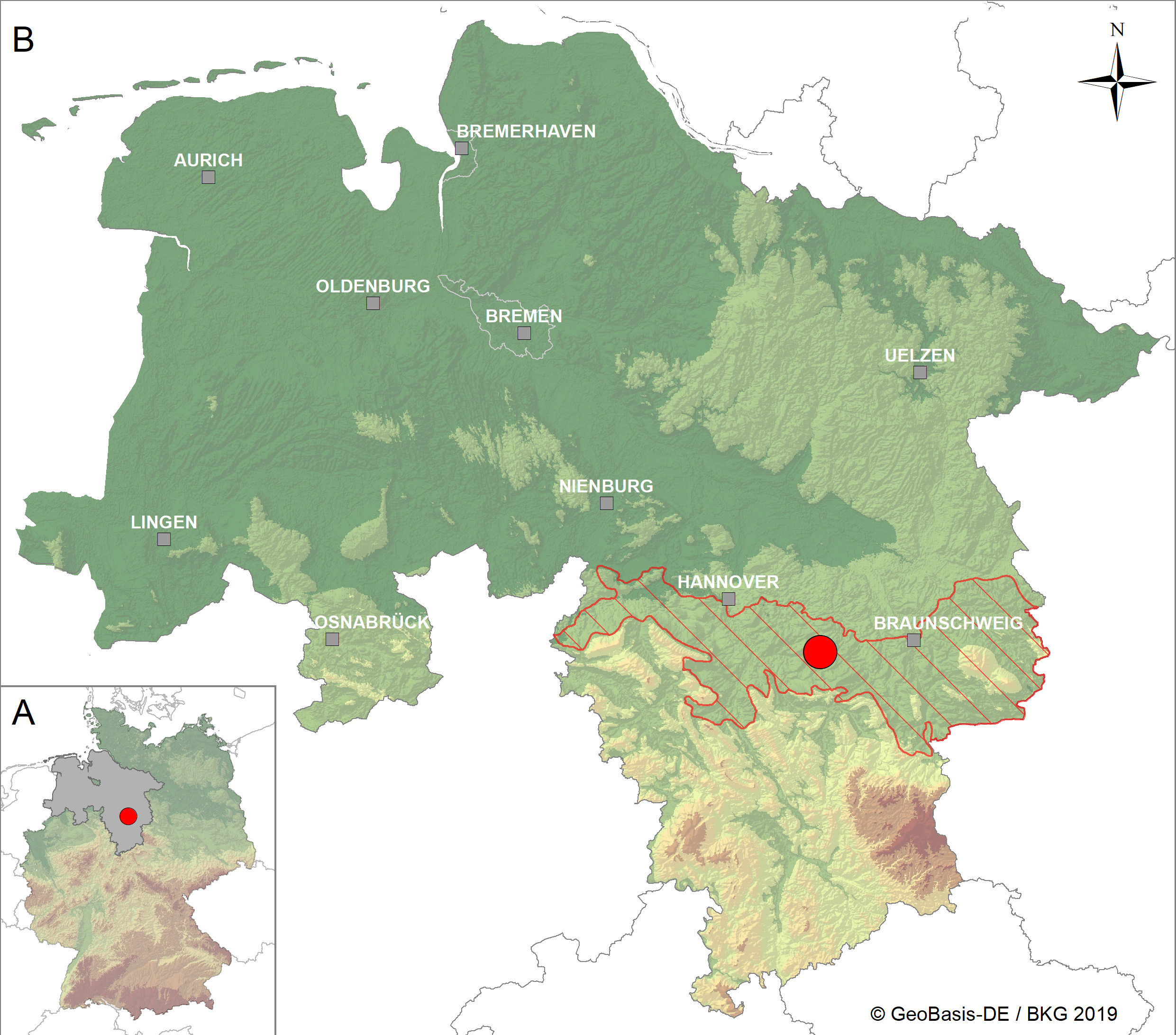

Supplement: S1 Fig — A) Base map with location of the study area (red dot) in the federal state Lower Saxony (grey shading) in Germany. B) The elevation map shows the geographic position of the study area (red dot) in the North German Plains. The study area is situated in the natural region ‘Hildesheimer Börde’ (red hatched area), which is characterized by an intensively used agricultural landscape. For orientation some cities (grey squares) are shown. (TIF) [file pone.0222205.s001.tif]

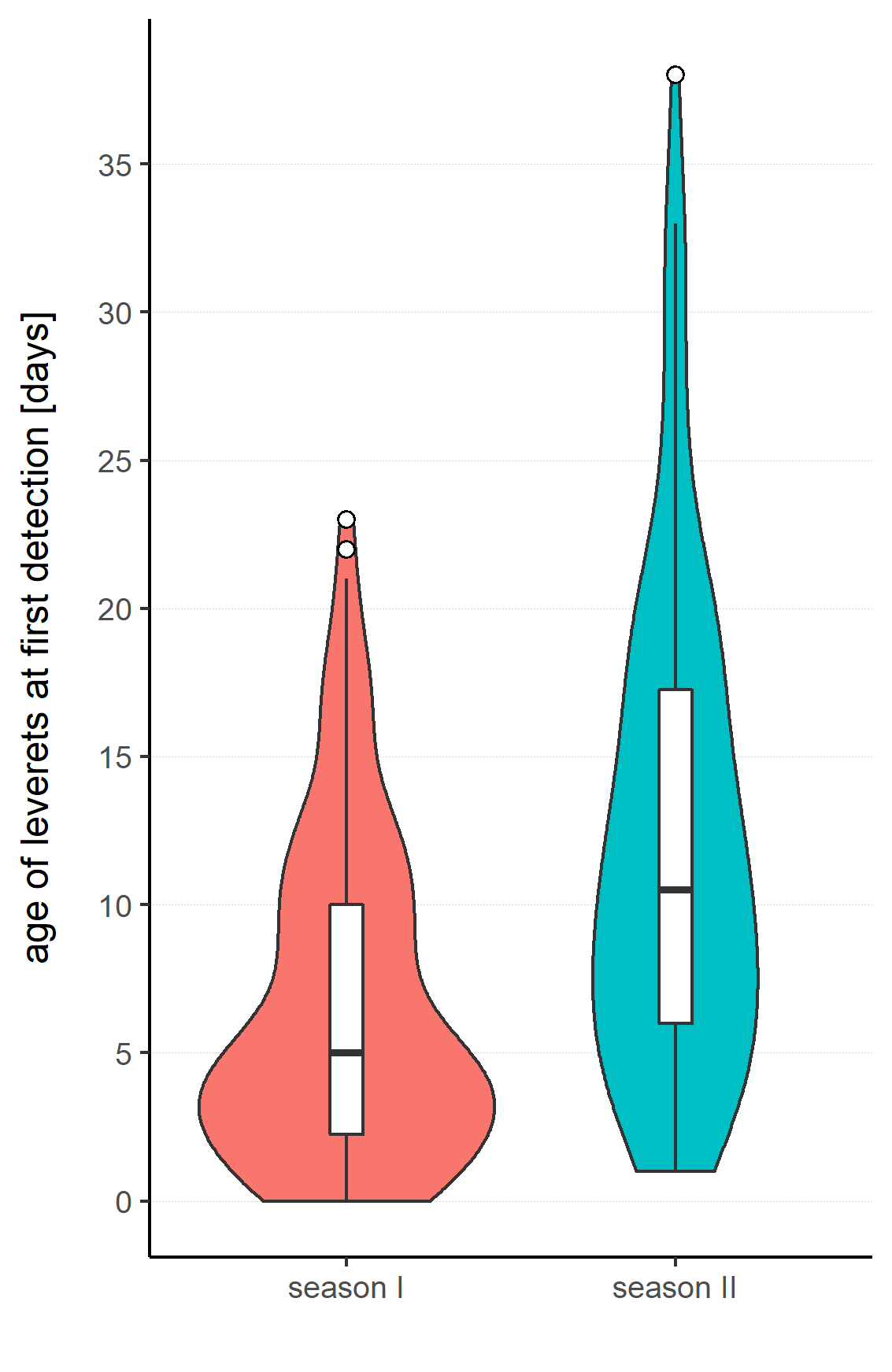

Supplement: S2 Fig — The violin and box plot prove that thermography is a useful tool for detecting leverets within their first days of life. 75% of all juveniles found were less than ten and 17 days old in season I (red violin = spring) and season II (blue violin = summer), respectively. (TIFF) [file pone.0222205.s002.tiff]
