# Supplementary material for: Living on the edge - circadian habitat usage in pre-weaning European hares (Lepus europaeus) in an intensively used agricultural area
Source: PLoS One. 2019 Sep 9;14(9):e0222205. doi: 10.1371/journal.pone.0222205 (PMC6733508; doi:10.1371/journal.pone.0222205)
Supplement: S1 Table — The type of use is classified as preference/occupancy (+), equal use/no selection (=) or avoidance/non-occupancy (-). (DOCX) [file pone.0222205.s003.docx]

S1 Table. Use-availability analyses for aggregated habitat classes and both seasons in the daytime.

| Habitat class | | Area (ha) | Percentage of area | Number of locations | Percentage observed | Expected numbers | Bailey’s 95% confidence limits | | Type of use |
| --- | --- | --- | --- | --- | --- | --- | --- | --- | --- |
|  |  |  |  |  |  |  | lower | Upper |  |
| C20 | crops 0-20 m | 187 | 0.210 | 26 | 0.056 | 97.81 | 0.02925 | 0.09185 | - |
| C60 | crops 20-60 m | 293 | 0.330 | 24 | 0.052 | 153.47 | 0.02612 | 0.08652 | - |
| C100 | crops 60-100 m | 182 | 0.205 | 20 | 0.043 | 95.15 | 0.02005 | 0.07568 | - |
| C>100 | crops >100 m | 118 | 0.133 | 9 | 0.019 | 61.69 | 0.00526 | 0.04411 | - |
| PA | pasture | 42 | 0.047 | 7 | 0.015 | 21.95 | 0.00312 | 0.03789 | - |
| RD | roadside ditch | 4 | 0.005 | 10 | 0.022 | 2.17 | 0.00642 | 0.04715 | + |
| RA | resid. assoc. | 6 | 0.007 | 9 | 0.019 | 3.06 | 0.00526 | 0.04411 | = |
| DG | ditches-grassy | 4 | 0.005 | 18 | 0.039 | 2.27 | 0.01711 | 0.07017 | + |
| FS | fallow-storage | 8 | 0.009 | 62 | 0.133 | 4.35 | 0.09121 | 0.18246 | + |
| CH | copses-hedges | 7 | 0.008 | 64 | 0.138 | 3.57 | 0.09485 | 0.18730 | + |
| TR | tracks | 37 | 0.042 | 216 | 0.465 | 19.50 | 0.39713 | 0.52991 | + |
|  | total | 888 | 1.000 | 465 | 1.000 | 465 |  |  |  |

The type of use is classified as preference/occupancy (+), equal use/no selection (=) or avoidance/non-occupancy (-).
