# Supplementary material for: Living on the edge - circadian habitat usage in pre-weaning European hares (Lepus europaeus) in an intensively used agricultural area
Source: PLoS One. 2019 Sep 9;14(9):e0222205. doi: 10.1371/journal.pone.0222205 (PMC6733508; doi:10.1371/journal.pone.0222205)
Supplement: S2 Table — The type of use is classified as preference/occupancy (+), equal use/no selection (=) or avoidance/non-occupancy (-). (DOCX) [file pone.0222205.s004.docx]

S2 Table. Use-availability analyses for aggregated habitat classes and both seasons at nighttime.

| Habitat class | | Area (ha) | Percentage  of area | Number of locations | Percentage observed | Expected numbers | Bailey’s 95%  confidence limits | | Type of use |
| --- | --- | --- | --- | --- | --- | --- | --- | --- | --- |
|  |  |  |  |  |  |  | lower | upper |  |
| C20 | crops 0-20 m | 309 | 0.204 | 336 | 0.381 | 179.98 | 0.33417 | 0.42786 | + |
| C60 | crops 20-60 m | 492 | 0.325 | 226 | 0.256 | 286.87 | 0.21501 | 0.29935 | - |
| C100 | crops 60-100 m | 309 | 0.204 | 85 | 0.096 | 179.72 | 0.06975 | 0.12719 | - |
| C>100 | crops >100 m | 218 | 0.144 | 31 | 0.035 | 126.89 | 0.01954 | 0.05594 | - |
| PA | pasture | 78 | 0.051 | 27 | 0.031 | 45.15 | 0.01617 | 0.05032 | - |
| RD | roadside ditch | 8 | 0.005 | 0 | 0.000 | 4.57 | 0.00000 | 0.00626 | = |
| RA | resid. assoc. | 10 | 0.006 | 3 | 0.003 | 5.65 | 0.00009 | 0.01294 | = |
| DG | ditches-grassy | 7 | 0.005 | 3 | 0.003 | 4.06 | 0.00009 | 0.01294 | = |
| FS | fallow-storage | 13 | 0.008 | 65 | 0.074 | 7.43 | 0.05046 | 0.10148 | + |
| CH | copses-hedges | 14 | 0.009 | 25 | 0.028 | 8.17 | 0.01452 | 0.04748 | + |
| TR | tracks | 58 | 0.038 | 81 | 0.092 | 33.51 | 0.06585 | 0.12209 | + |
|  | total | 1514 | 1.000 | 882 | 1.000 | 882 |  |  |  |

The type of use is classified as preference/occupancy (+), equal use/no selection (=) or avoidance/non-occupancy (-).
